# Implementation of a Statewide Web-Based Caregiver Resource Information System (CareNav): Mixed Methods Study

**DOI:** 10.2196/38735

**Published:** 2022-07-13

**Authors:** Heather M Young, Janice F Bell, Orly Tonkikh, Tina R Kilaberia, Robin L Whitney, Jennifer M Mongoven, Benjamin M Link, Kathleen Kelly

**Affiliations:** 1 Family Caregiving Institute Betty Irene Moore School of Nursing University of California Davis Sacramento, CA United States; 2 The Valley Foundation School of Nursing San Jose State University San Jose, CA United States; 3 Family Caregiver Alliance San Francisco, CA United States

**Keywords:** online assessment, caregiver, technology implementation, Consolidated Framework for Implementation Research, CFIR

## Abstract

**Background:**

With the aging population, family caregivers provide increasingly complex and intense care for older adults and persons with disabilities. There is growing interest in developing community-based services to support family caregivers. Caregiving occurs around the clock, and caregivers face challenges in accessing community-based services at convenient times owing to the demands of care. Web-based resources hold promise for accessible real-time support. CareNav (TM), a caregiver resource information system, is a web-based platform designed to support real-time universal caregiver assessment, a record of client encounters, development of a care plan, tailored information and resource content, access to web-based caregiver resources, the capacity to track service authorization and contracts, and secure communications. The assessment includes needs and health conditions of both the care recipient and caregiver; current resources; and priorities for support, information, and referral. In 2019, the California Department of Health Care Services funded the 11 nonprofit California Caregiver Resource Centers (CRCs) to expand and improve family caregiver services and enhance CRC information technology services. Deployment of a statewide information system offered a unique opportunity to examine structures and processes facilitating implementation, providing feedback to the sites as well as lessons learned for similar projects in the future.

**Objective:**

The aim of this paper was to describe the statewide implementation of the comprehensive CareNav system using the Consolidated Framework for Implementation Research as an organizing structure for synthesizing the evaluation.

**Methods:**

This mixed methods study used two major approaches to evaluate the implementation process: a survey of all staff who completed training (n=82) and in-depth qualitative interviews with 11 CRC teams and 3 key informants (n=35). We initially analyzed interview transcripts using qualitative descriptive methods and then identified subthemes and relationships among ideas, mapping the findings to the Consolidated Framework for Implementation Research.

**Results:**

We present findings on the outer setting, inner setting, characteristics of the intervention, characteristics of the staff, and the implementation process. The critical elements for success were leadership, communication, harmonization of processes across sites, and motivation to serve clients in more accessible and convenient ways.

**Conclusions:**

These findings have implications for technology deployment in diverse community-based agencies that aspire to enhance web-based services.

## Introduction

### Background

In the United States, approximately 20% of adults provide unpaid care to a family member or friend [1]. Family caregivers provide the vast majority of long-term care (valued at US $470 billion annually), eclipsing annual governmental spending on long-term care at US $430 billion [2]. Caregiving is often a commitment spanning years and a variety of domains including personal assistance, instrumental aid and emotional support, care coordination, and managing chronic health conditions. With shortened hospital stays, family caregivers now perform complex health care including medical or nursing tasks previously within the purview of health care professionals [3]. Despite the common experience of caregiving, few individuals are prepared for the demands of the role [4]. Many people experience strain, depression, loneliness, deterioration in their own health, and financial distress in the course of providing care [1]. Even so, most are unaware of the existing resources to support them.

Despite their vital role in optimizing function and health for older adults, caregivers are relatively invisible in health care. Caregivers provide valuable information about the person receiving care, yet rarely do health care professionals assess the capacity, readiness, or mental health of caregivers to provide care during routine health encounters [3,5].

### Web-Based Supports for Caregivers

In the absence of routine caregiver assessment and support in the clinical setting, there has been growing interest in developing community-based services to support family caregivers. Caregiving occurs around the clock, and caregivers face challenges in accessing community-based services at convenient times owing to the demands of care. Web-based resources hold promise for accessible real-time support, and targeted interventions have been developed for specific audiences such as caregivers for persons with cancer [6], dementia [7-10], and other chronic conditions [11-13].

Most web-based supports emphasize one element such as psychoeducational offerings or stress management. Despite quality issues with many studies, the positive outcomes of targeted interventions have included improved self-efficacy, improved self-esteem, and less strain [14]. Missing from existing web-based interventions are a global assessment of the needs of the caregiver and care recipient and the ability to respond to the priority of the caregiver at the time, whether it is for information, referral to specific local services, or counseling.

Studies on web-based caregiver support have demonstrated 3 tendencies. First, caregivers of persons with dementia are overrepresented because of the demanding nature of caregiving related to issues with memory, thinking, and behavior. Many studies on web-based support for dementia caregivers, such as iSupport [10], focus on psychoeducational interventions that help caregivers cope with stress, anxiety, caregiving burden, quality of life, awareness of stressors and needs, and caregiving competence. Overall, studies of such psychoeducational interventions have reported improvements in the well-being and preparedness of caregivers [15-17]. However, the effect size is medium-small even in randomized controlled trials and quasi-experimental studies [15]. Significant variations in content, structure, outcome measures, and intervention duration further prevent cogent conclusions within web-based support studies for caregivers [18].

Second, the heterogeneity of web-based support types underlies varying results. Studies on web-based support for those caring for persons with cancer [19], posttraumatic stress disorder [20], and psychosis [21,22] offer findings that reflect varied web-based support needs for different caregivers. Disease type, intervention type, dosage (amount of time spent on the web), and duration affect the effectiveness, feasibility, and quality of interventions [23]. The navigation and intuitiveness of web-based sessions may depend on the distinct needs of caregiver subgroups [22].

Third, the breadth and scope of caregiver support in the web-based modality affords subgroup-specific knowledge rather than exhaustive knowledge on implementation strengths and challenges. For example, in an implementation study of a video health technology intervention to improve self-care of caregivers of persons with heart failure, Hirschman et al [24] found adaptation challenges related to hardware, software, and network connectivity. In a behavioral intervention for dementia caregivers that had significant improvements for caregivers, Nichols et al [16] discerned as important the clinical success, leadership and staff support, and ongoing need for modifications while maintaining fidelity, linkage to the organizational context, and fiscal health. In mapping directions for research, Lindeman et al [25] underscored, among others, matters of equity, inclusion, and access; privacy and security; and the influence of political and regulatory factors on interoperability. To date, research has not examined broad-based web-based resources that include caregivers involved across different health conditions and provide a full array of supports, from information and referral to individual and group support, to legal and respite service provision. The aim of this paper was to describe the statewide implementation of the comprehensive CareNav (TM) system using the Consolidated Framework for Implementation Research (CFIR) as an organizing structure for synthesizing the evaluation.

### Supporting Caregivers in California

CareNav, a caregiver resource information system, is a web-based platform designed to support interactive universal caregiver assessment, a record of client encounters, development of a care plan, tailored information and resource content, access to web-based caregiver resources, capacity to track service authorization and contracts, and secure communications. The assessment includes both care recipient and caregiver needs and health conditions; current resources; and priorities for support, information, and referral. The assessment can be self-administered on the web or administered by a staff member over the phone or in-person. After assessment, a staff consultant meets with the caregiver to prioritize and develop a care plan that might include contracting services such as respite, referral to educational offerings or a support group, or vouchers for legal aid or counseling.

The Family Caregiver Alliance (FCA), one of the 11 California Caregiver Resource Centers (CRCs; California State System of Support for Caregivers), pioneered CareNav with private funding and deployed this system across 3 CRCs that served as pilot sites. In 2019, the California Department of Health Care Services funded 11 nonprofit CRCs to expand and improve family caregiver services and enhance CRC information technology services, deploying CareNav as a common data set across the state over a 3-year period (2019-2022).

The FCA led the implementation team for CareNav in partnership with the technology developer Quality Process (QP). The FCA contracted with the University of California Davis Family Caregiving Institute to conduct an evaluation of the implementation process as well as an analysis of the statewide data to determine program effectiveness and quality improvement opportunities. The implementation began in January 2020, and the system was fully deployed by September 2020.

### Organizing Framework

**Figure 1:**
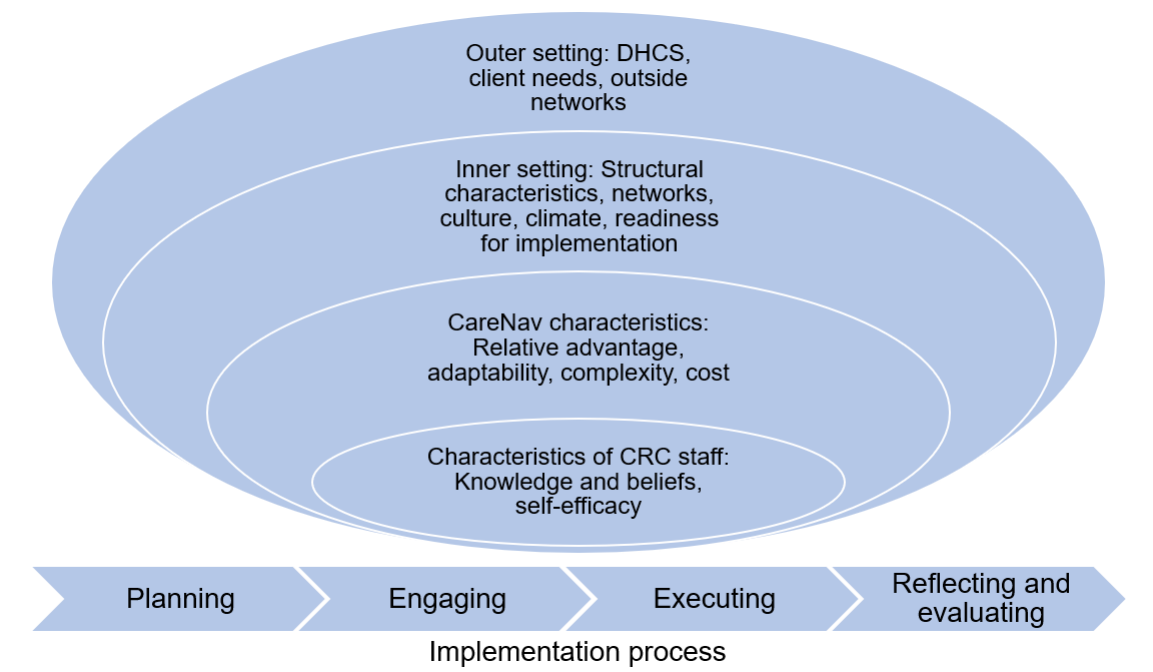
The Consolidated Framework for Implementation Research model for CareNav implementation. CRC: Caregiver Resource Center; DHCS: Department of Health Care Services.

Implementation evaluation was guided by the health-focused CFIR [26]). The CFIR includes five domains that organize our mapping of the results (Figure 1): the outer setting, the inner setting, the intervention characteristics, staff characteristics, and the process of implementation. The outer setting includes the social and economic context within which the statewide CRC system resides, particularly considering relationships with outside organizations, client needs, and the effects of the COVID-19 pandemic. The inner setting refers to the levels and characteristics of the organization, in this case the CRC statewide system, focusing on structural characteristics, culture, the implementation climate, networks and communications, and readiness for implementation. The intervention characteristics acknowledge the perception of the stakeholders about the key attributes of CareNav including relative advantage, adaptability, complexity, and cost. The characteristics of individuals recognize staff knowledge and attitudes toward CareNav as well as beliefs about the capabilities required for the implementation and stage of change. The implementation process considers 4 major aspects: planning, engaging, executing, and reflecting and evaluating [26].

## Methods

### Design Overview

This mixed methods study used two major approaches to evaluate the implementation process: in-depth interviews with key informants at the CRCs, and surveys of all staff who completed training. The in-depth interviews explored all aspects of the implementation process from multiple perspectives, while the surveys were used to characterize the readiness and self-efficacy of the staff implementing the system. This approach will enable the evaluation team to assess readiness and satisfaction over time using quantitative scores while developing a deeper understanding of the dynamics of change and appreciation of multiple perspectives afforded by qualitative interviews.

### Qualitative Interviews

The evaluation team conducted focus group interviews with teams of each CRC, and individual interviews with key informants from the implementation team. All current leaders and staff of the 11 CRCs were eligible to participate in this study. A focus group was established for each CRC to include all interested staff from the same site. The interviews elicited perspectives on implementation and training activities, including perceptions of benefits and concerns regarding CareNav. We used a semistructured interview guide for both focus groups and individual interviews asking about the implementation process, challenges and facilitators, anticipated system and client outcomes, and satisfaction with the process. Owing to the widespread implications of the COVID-19 pandemic on service delivery, we asked all participants about how the COVID-19 pandemic affected both the implementation process and experiences of their clients as caregivers. Interviews were conducted using Zoom, and audio was recorded with the consent of the participants and transcribed.

### Pretraining and Posttraining Surveys

The evaluation team designed a pretraining and posttraining readiness survey to determine readiness, preparation, and confidence regarding the implementation process and to identify self-efficacy and perceived benefits and concerns before and after training. We invited all staff to complete surveys before and immediately after a full day of formal training on CareNav.

The pretraining readiness survey included 10 items rated on a 5-point scale, where 1 represents the most positive response. Cronbach α for the current sample was .83. The survey also assessed whether the participants are familiar with CareNav, know its purpose, and how to do an intake and assessment. Open-ended questions identified benefits and concerns about CareNav.

The posttraining survey reassessed participants’ knowledge, preparedness for implementation, confidence, and knowing where to get help with CareNav. The posttraining survey also assessed whether the training met participants’ needs and their willingness to take actions that could support CareNav implementation, such as encouraging staff or coworkers to use CareNav and ensuring new staff members are educated on how to use CareNav.

### Data Analysis

The transcribed interviews were imported into the Dedoose qualitative data analysis software. Qualitative descriptive methods were used to analyze the transcripts and open-ended responses to the surveys [27,28]. Two members of the research team (HMY and TRK) reviewed the transcripts and developed initial codes and definitions. They independently coded the same transcript and then compared coding decisions, refined definitions, and arrived at a consensus about coding and documenting changes to the definitions. All the transcripts were subsequently coded into agreed-upon general categories. A second analysis (by HMY and OT) identified the subthemes and relationships among ideas, mapping the findings to the CFIR.

Quantitative data were analyzed using descriptive statistics. To enable meaningful interpretation and visualization, the 10-item readiness scale was recoded to a 5-point scale in which a higher score represents better readiness. Pretraining and posttraining scores were compared using paired 2-tailed *t* tests for continuous variables and McNemar tests for dichotomous variables. Quantitative analyses were performed using the SPSS statistical package (version 27; IBM Corporation).

### Ethical Considerations

This study was determined to be exempt from ethics approval by the UC Davis institutional review board. We collected no identifying information about the participants in the survey and focus groups. Participants were informed about the purpose of the study and the voluntary nature of participation, providing assent by completing the survey or continuing with the recorded Zoom interview.

## Results

### Participants

#### Pretraining and Posttraining Readiness Surveys

In total, 86 staff members completed training, with 82 (95%) participants contributing pretraining data and 56 (65%) participants contributing posttraining data. The staff included CRC directors, family consultants who interact with caregiver clients and provide resources and support, and analysts who manage client and financial data. The surveys were anonymous, with no identifying data of the staff members. The results of readiness and self-efficacy are presented in subsequent sections within the CFIR model as characteristics of the staff.

#### Qualitative Interviews

Between May and August 2020, we conducted 11 focus group interviews (ranging in size from 2 to 6 participants) and 5 focused individual interviews with key informants, totaling 35 participants. Participants represented all 11 sites and the implementation team and included all roles (directors, clinical, and technical staff). To protect the privacy of the participants, we did not collect demographic data associated with these interviews.

### Evaluation of Implementation

We mapped the findings of this evaluation to the following CFIR domains: outer setting, inner setting, characteristics of the intervention, staff characteristics, and implementation process. The specific findings related to these domains have been expanded upon in the subsequent sections.

### Outer Setting

**Figure 2:**
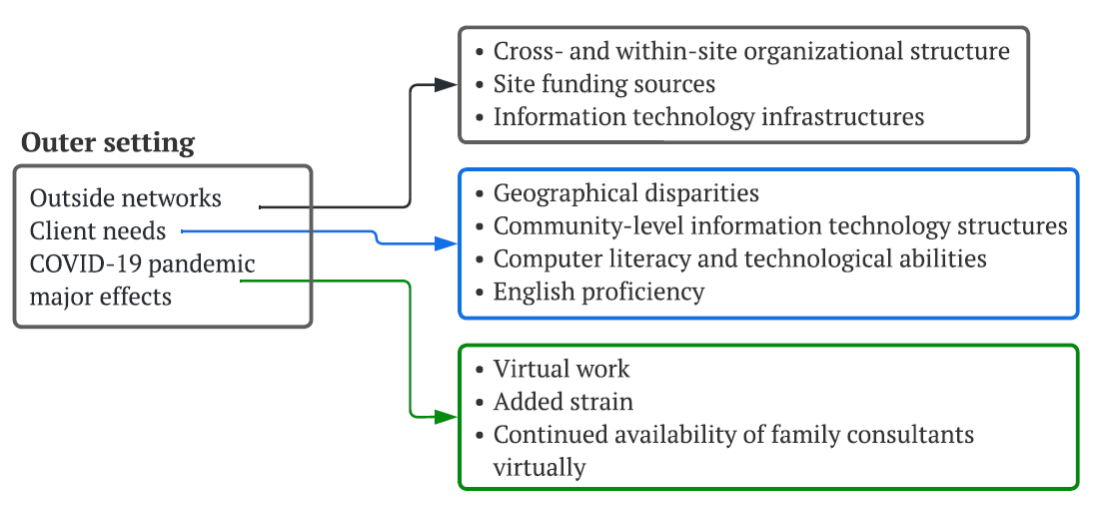
Outer setting.

Interviews across CRC sites revealed site-level variability and heterogeneity in relationships and networking with external organizations as well as the diversity of client characteristics and needs (Figure 2). Implementation occurred during the COVID-19 pandemic, shaping additional contextual factors.

#### Outside Network

Some CRCs exist as standalone organizations. More complex sites are embedded or hosted in larger health care systems with their own information technology platforms, which requires additional efforts to integrate CareNav and to be compliant with additional health system privacy and security policies. Moreover, the funding sources and constellations of services vary between sites and influence both documentation and administrative requirements. This challenged the transition from the existing local databases to the CareNav platform. For example:

Because it’s a hospital it has security really well locked down. And so, the website that I need to access to see if the data is correct in their initial upload in our system is blocked. So, I’m working within my own organization to try to get that website unblocked.

#### Client Needs

Geographic disparities are reflected in rural sites serving communities that lack the adequate technology structures and broadband connection necessary for reliable access to an internet platform. Clients are also diverse in their English proficiency and their capacity to use technology. Several participants mentioned low computer literacy and lack of technological and internet safety skills as barriers for older caregivers to use technology. Nonetheless, some of the staff reported being surprised by the amount of participation by clients whom they assumed would not ordinarily use technology. This observation was partially attributed to the consequences of the COVID-19 pandemic:

But I think a lot of them are open to technology. I think this pandemic has forced that issue where a lot of family caregivers are using more technology now...And I'm surprised as to the ones that I thought wouldn't know how to use technology. The ones that before COVID there were a lot that I never, never thought that they could do it as a video with me. I never thought that because I just, you generalize people...because of age...a lot of them are facetiming family, they are video conferencing with their family care navigator...so we just have to start thinking differently as far as how we get them used to CareNav.

#### COVID-19 Pandemic: Major Effects

The COVID-19 pandemic, which emerged simultaneously with the implementation kickoff, amplified the existing risks and threats to the well-being of caregivers. Family consultants reported higher levels of stress, more financial concerns, increased housing and food insecurity, job loss, and escalating costs of caregiving supplies (such as gloves and masks) among their clients. The strain was exacerbated by loss of support resources, including availability of adult day care, home help, in-person support groups, and a reluctance to consider assisted living or skilled nursing alternatives at this time as residential care facilities were widely reporting COVID-19 outbreaks and, in some cases, higher mortality rates for older persons. Many caregivers faced additional home demands, caring for multiple family members and children being schooled at home. The pandemic increased their sense of isolation, with family consultants reporting more symptoms of depression and anxiety among their clients. Finally, caregiver health was further compromised by the inability to quarantine when a family member was ill, thus increasing the exposure of the caregiver. Caregivers also expressed reluctance to access formal services owing to fear of contagion or not having the time to devote to their own health:

We’ve seen kind of that increased risk, I think, across the board because a lot of our support systems are not available right now. [...] I think what most impacts caregivers, or can impact their health overall and wellbeing, is what level of support they have available to them.

The pandemic created a particularly challenging climate for the implementation of a new major system***.*** Additional strain was placed on staff members who were already facing a learning curve to execute the new system, as well as dramatic changes in their workflow. Simultaneously, it accelerated the need for a web-based platform and rapidly demonstrated the usefulness and feasibility of the web-based environment (eg, continued availability of family consultants virtually). Interview participants highlighted how the pandemic demonstrated the benefits of CareNav:

I think the Coronavirus kind of shows how helpful it is to have everything online. There really hasn’t been too much of a disruption to the services we’re able to provide our caregivers. I think that’s been a huge plus to be able to see how nicely it can transition when things do happen, that we’re still able to access what we need.

### Inner Setting

**Figure 3:**
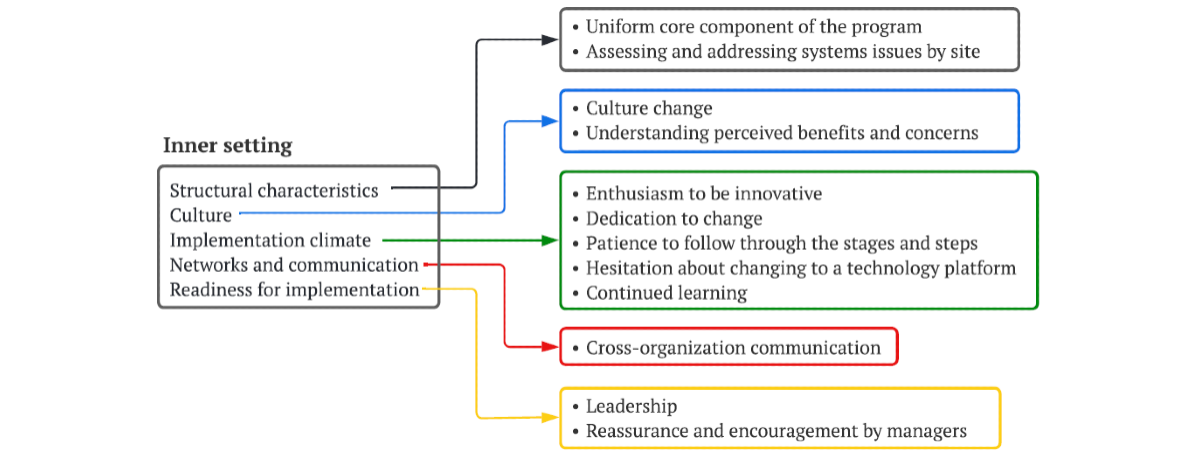
Inner setting.

The inner setting for CareNav implementation is the state-level CRC system and 11 individual CRCs (Figure 3).

#### Structural Characteristics

California launched a CRC system in 1984 with the enactment of the Comprehensive Act for Families and Caregivers of Brain-Impaired Adults, thereby establishing a network of support for caregivers. At present, the California Department of Health Care Services (DHCS) funds 11 CRCs that provide support to family caregivers affected by chronic health conditions, including Alzheimer disease and related dementias and other degenerative diseases.

CRCs serve as a point of entry for services available to caregiving families, covering every county in California. While each center tailors its services to its geographic area, all CRCs have a core component of programs that provide uniform caregiver assessment and information, education, and support for caregivers. Individual CRCs also receive funds from county contracts, foundations, business partners, and donations to provide additional services. For over three decades, CRCs have supported caregivers in their regions, relying on staff intake interviews with caregivers and tailored referrals to relevant resources. With fluctuating funding, the programs became decentralized, and over the past decade, the 11 CRCs had been operating relatively independently. Funding to implement the statewide web-based platform provided an opportunity for new collaborations and connections.

#### Culture

CRCs provide services across income categories, and the original enabling legislation included middle-income families who are often overlooked and for whom few services are targeted. CRCs are united by shared values emphasizing choice, collaboration, innovation, quality, participation, respect, and diversity.

The implementation prompted culture change in three major ways: formalizing the system of CRCs across the state by uniting loosely affiliated sites, instituting standardized assessments, and changing to a new way of delivering services to clients virtually. The funding and subsequent process fostered a shared goal among CRC directors to serve the entire state and collaborate with one another to do so. Committing to a shared technology platform involved greater discussion and information exchange among the groups as well as the recognition that the whole is greater than the sum of the parts. A welcome culture change was the comradery and mutual aid that solidified over the initial year of implementation:

I feel like just within the CRC system the genuine enthusiasm for the project. Just to embrace it, even with all this other kind of craziness [COVID-19 pandemic] going on in the world.

#### Implementation Climate

In-depth interviews revealed a collective positive attitude with a willingness to change, staff dedication, continued learning, and patience to follow through the stages and steps of the process. All interview participants expressed enthusiasm for the adoption of CareNav and a belief in the positive potential of this change in their practices:

I think it's been well received. We've been anticipating this for a long time. And have been very excited about it...We have some folks that were really excited and got right in there and started playing with it and working with it. Others I think are a little bit, you know, we're learning as we go.

Participants expressed hesitation about changing to a technology platform but recognized that caregivers’ needs are changing in society and that the systems must evolve to meet the changing needs.

The idea is to be innovative and for caregivers, because, as things continue our younger population is very used to being more self-directed and being online. So, it has potential for growth and change over time. We'll continue to do that with a lot of streamlining of systems.

#### Networks and Communication

Communication was vital at all levels, from the implementation team to the sites, from the directors to their staff, and among sites. Communication involved developing a shared vision for the process and the outcomes, as well as coordinating the logistical aspects of the implementation. The process of preparing for technology deployment revealed workflow and processes that were not initially evident or taken for granted, and these had to be addressed as technology was applied to automate processes. Communication among the teams was vital to understanding work processes and establishing new ways of operating as needed.

Communication and support from FCA and among CRC sites played a major role in successful implementation, particularly as sites faced delays or barriers and were able to benefit from lessons learned elsewhere:

Something that's new is now we are communicating and reaching out to the other CRCs, which I believe hasn't happened in years. So it's really great that if we have a question or wondering, a different way of doing something in our program, I can just reach out and to anyone you know, in California and get their help and opinion.

Both the implementation process and the shared platform facilitated deeper collaboration among sites and the ability to elevate local issues with colleagues across the state, enhancing the strength of recommendations to address caregiver needs more comprehensively.

#### Readiness for Implementation

At the beginning of the implementation effort, 3 CRC sites, including FCA, were already using CareNav. The subsequent sites benefited from reliability and usability testing, as well as refinements and improvements that had been made during the initial, more limited deployment by FCA and QP.

Leadership was an essential condition for success from the implementation team and at the CRC site level. Strong leadership, reassurance, and encouragement by managers, coupled with effective communication, established the overall vision, a shared understanding of the goal, the anticipated process and outcomes, and motivated staff across the sites to engage in implementation:

I think there's been leadership behind this and a sense of vision...FCA is [a] very unique organization and that [sic] they are really sitting in this point where they're both policy wonks as well as clinical experts. And I think that this allows FCA to bring vision and be forward looking of where we're going. And I think that the other CRCs are benefiting from that.

### CareNav Characteristics

**Figure 4:**
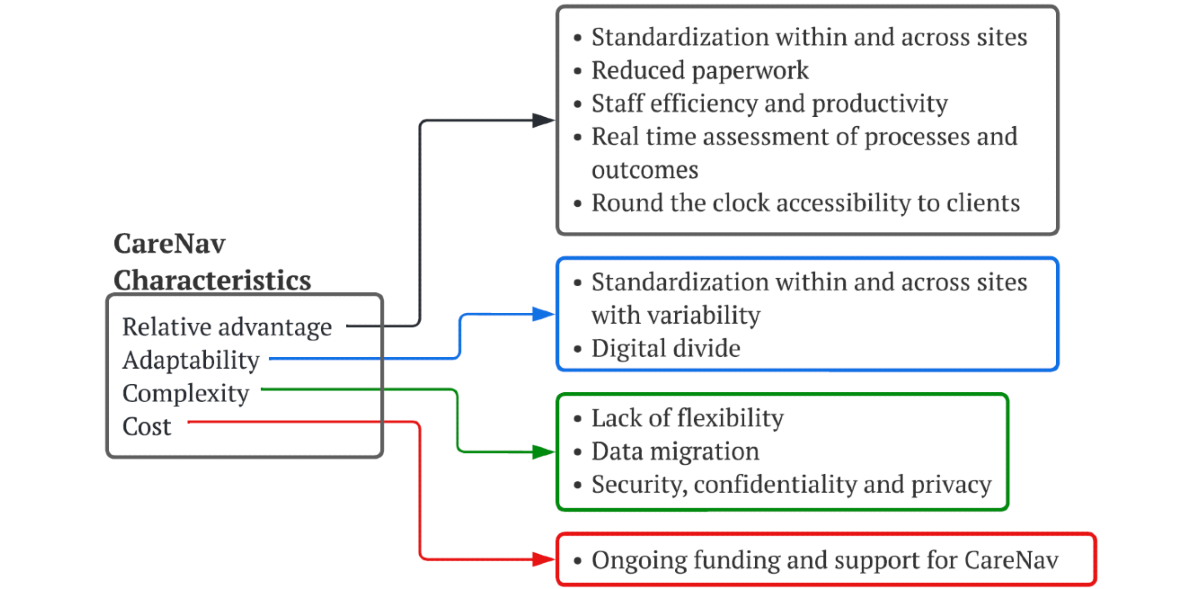
CareNav characteristics.

Focused interviews point to CareNav attributes that both facilitate and inhibit its implementation (Figure 4).

#### Relative Advantage

A key factor for the intervention is its relative advantage over previous data collection tools and software used by CRC sites to gather and aggregate site-level data on the caregivers they served and the programs they administered. Using CareNav reduced paperwork, enabled easier documentation, more efficient and secure charting, and a more environment-friendly approach. Additional advantages included increased CRC capacity and round-the-clock accessibility for clients. Several participants emphasized how the design of CareNav supports the process of care:

Now when a new person starts it’s...here’s the computer system that we use. This is the workflow, because I think it's really laid out nicely, the toolbar at the top of care journey. There’s a workflow that you follow. It’s just all right there in one place and as long as you have a secure connection, you can access it, so it’s going to save a lot of time. I think people are going to end up really happy that they have that information at their fingertips, and they don’t have to write it down on paper or search for it, laboriously, in their client file.

Both clinicians and managers reported the advantages of CareNav for management functions. Standardization of the system across CRCs was perceived as allowing sites to collaborate for consultation, evaluation, and advocacy. Using a uniform tool supports administrative and management functions, including case data record-keeping as well as assessing the process and outcomes of the organization in real time:

For a manager being able to pull up reports without it being having to dump it into Excel and then sort through all the different things that you don’t need. It looks like data collection is just going to be a lot simpler and easier...as a manager it’s easier to measure staff productivity on a system like that because you can go in their notes.

#### Adaptability

The most frequently identified benefit and concern about CareNav implementation is related to the adaptability of CareNav to the outer-setting variability among sites and populations served. Sites varied along multiple dimensions including population served, geographic characteristics, funding sources, relationships with host organizations, and size. The ability of CareNav to provide remote access, particularly in rural areas, was perceived as a benefit even with the variability in geographic distribution of clients in several CRCs and the impact of the COVID-19 pandemic.

Nonetheless, the digital divide (the issue of equitable access to technology and broadband connection) constitutes an ongoing barrier, particularly for rural, low-income, and non–English-speaking clients. Moreover, there are some caregivers in remote areas who value self-sufficiency and view government programs with suspicion, with reluctance to share information on the web regardless of access to broadband connection. Some communities in the catchment area do not have reliable broadband connections, and many clients may not be able to afford internet services or the associated technology. The platform is provided in English, limiting access to caregivers who do not speak English or have low literacy:

But language for me is probably my biggest concern, because we do have a big, Spanish speaking population and Vietnamese. It’s a very significant group.

#### Complexity

Sites hosted within a larger health care and information system experienced difficulties associated with the lack of flexibility and data migration, adding complexity and increasing the time and effort demands for staff. For instance, double data entry was necessary for some CRCs to ensure timely and accurate data for fiscal reporting and reimbursement across various funding agencies.

Confidentiality presents another level of complexity. While CareNav is a secure and private system, there is a learning curve for staff to understand the inherent privacy and security protection of the care team and clients. To assure a secure environment, written direct communication with clients changed with CareNav, requiring user authentication processes to maintain clients’ privacy:

In the beginning, one of the issues that I was having, for example, when I was doing intake, if I sent emails, I was sending emails through CareNav. But people were not replying back and it turns out because it would send them a message into their actual email, but it would go to Spam....People just prefer for me to directly email them to their email.

#### Cost

The threat of delay in funding and losing funding during the budget negotiations raised concerns about ongoing funding and support for CareNav. Several sites expressed frustration with the delays associated with state contracting and system readiness. Time-limited funding without a clear path to sustainability posed challenges for directors in their long-term planning.

### Characteristics of the CRC Staff

#### Overview

**Figure 5:**
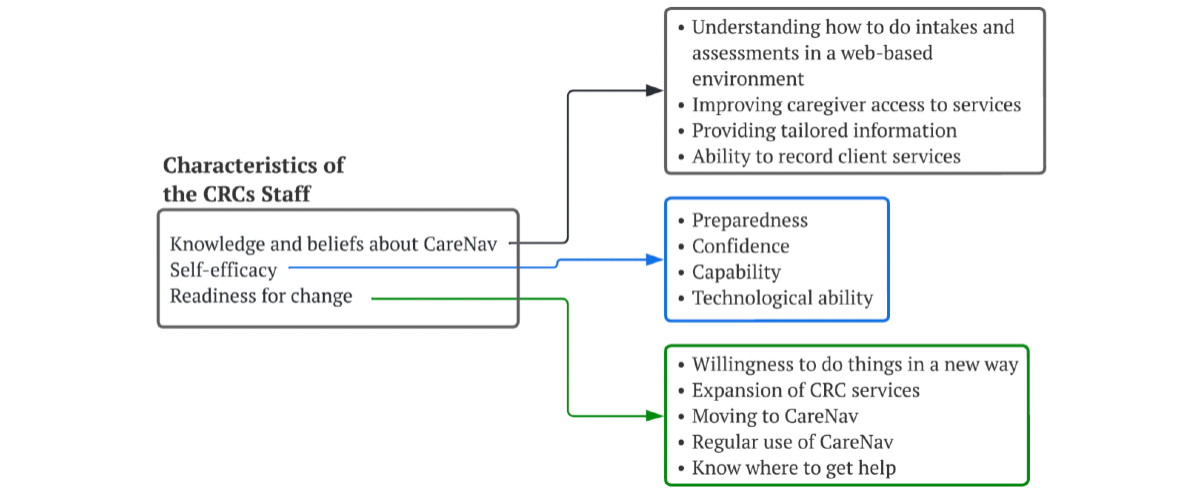
Characteristics of the Caregiver Resource Center (CRC) staff.

The pretraining survey was an early opportunity to evaluate CRC staff knowledge about CareNav, self-efficacy, and readiness for change (Figure 5). Overall, the participants had very positive attitudes toward the implementation of CareNav (Table 1), with a total readiness score of 3.8 (SD 0.6) and average responses to almost all items in the positive range.

**Table 1:** Pretraining Readiness Survey (N=82).

Item		n (%)
**Knowledge and beliefs about CareNav**		
	Seen or heard about CareNav^a^	73 (89)
	Understand how to do an intake and assessment in CareNav^a^	30 (37)
	CareNav is designed to improve caregiver access to services^a^	79 (98)
	CareNav will improve the ability to record services^b^	64 (74)
	CareNav provide tailored and accessible information for caregivers^b^	59 (68)
**Self-efficacy**		
	Prepared to implement CareNav^c^	43 (49)
	Confident to implement CareNav^c^	73 (84)
	Capable to implement CareNav^c^	59 (68)
**Readiness for change**		
	Positive with the expansion of CRC services^c^	71 (82)
	Positive with moving to CareNav^c^	66 (76)
	Willing about doing new things^c^	72 (83)
	It will take time to ensure regularly use of CareNav^b^ by everyone on the staff	73 (90)
	Know where to obtain help^b^	47 (54)

^a^Staff members reported “yes.”

^b^Staff members reported “strongly agree” or “somewhat agree.”

^c^Staff members reported “very positive/willing/prepared/confident/capable” or “somewhat positive/willing/prepared/confident/capable.”

#### Knowledge and Beliefs About CareNav

Almost 90% (73/82) of staff had seen or heard about CareNav before the training and almost all (79/82, 98%) believed that CareNav is designed to improve caregiver access to services. Before training, one-third (30/82, 37%) of the staff members reported understanding how to complete the intake and assessment in CareNav. The staff members also believed that CareNav would enable better structure and process of care including improved ability to record services (mean 4.3, SD 0.9) and providing tailored and accessible information for caregivers (mean 4.0, SD 0.9).

#### Self-efficacy

The staff expressed a high level of self-efficacy to implement CareNav in terms of feeling prepared (mean 3.3, SD 1.2), confident (mean 4.5, SD 0.9), and capable (mean 3.9, SD 1.2).

#### Readiness for Change

Regarding readiness for change, participants expressed a strong willingness to do new things (mean 4.5, SD 0.7) but believed that it would take time for everyone on the staff to become familiar with using CareNav (mean 1.5, SD 0.8).

### Implementation Process

**Figure 6:**
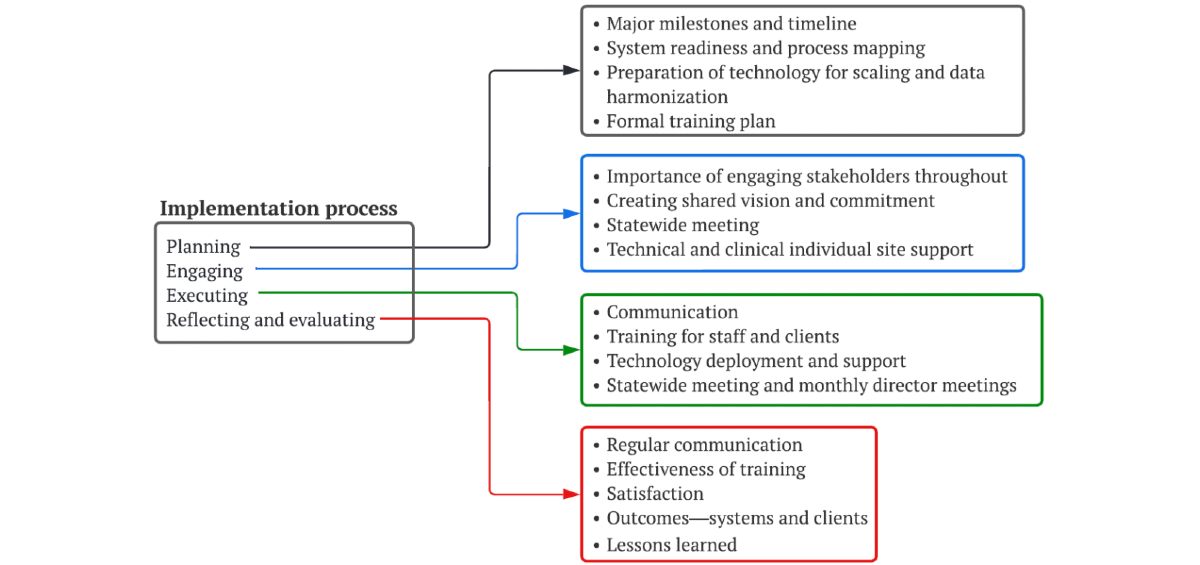
Implementation process.

The elements of implementation included developing an overall project plan, engaging stakeholders, preparing technology for scaling, general training, and providing technical expertise and support at each CRC site (Figure 6).

#### Planning

The implementation team consisting of staff from the FCA and QP developed a project management plan that addressed culture change, establishing site readiness, staff training, and technical implementation and support.

##### System Readiness and Process Mapping

Building on the demonstrated success and utility of CareNav in 3 sites, the implementation process involved planning for scaling the technology across 8 additional sites. As described in the inner and outer setting sections, the sites were diverse in several respects: the clients they served, the constellation of services and funding sources, and their relationship with a parent organization providing information technology. Early preparation involved assessing the requirements of each system for technology compatibility and interoperability, security, and compliance with applicable laws (eg, the Health Insurance Portability and Accountability Act). The technology team engaged with each site in mapping the data processes, identifying data sources, and reporting requirements.

##### Preparing the Technology for Scaling

The overall project plan involved determining major milestones along with the requisite resources and coordination to ensure progress. A major task of the technology team was to prepare the technology for scaling by harmonizing the data across the sites and mapping source data fields to the CareNav platform to assure standardization and data integrity. As the sites reviewed the CareNav software, they requested customization to fit their particular programs, funding requirements, and workflow, thus requiring site-specific revisions to the platform.

#### Engaging

The FCA team recognized the importance of engaging stakeholders throughout the process. They appreciated that scaling CareNav involved cultural change and made significant efforts to create a shared vision and commitment to engage in a new way. In a statewide kickoff meeting in January 2020, directors and staff of the CRCs came together to build relationships, develop a deeper understanding of CareNav and its deployment, and generate excitement regarding the effort. CRC sites shared best practices with one another and began to develop a stronger sense of collective resources and commitment to meet the needs of diverse caregivers across California.

General training was accomplished through an initial in-person kickoff meeting followed by statewide webinars. The webinars were widely attended and offered topics to address overarching issues, such as managing change, using telehealth and technology-enabled assessment and supports, and using data for quality improvement.

Technical and clinical support was provided by individual site-level trainings to prepare the staff for technology deployment, addressing site-specific workflow and learning needs. Starting in March 2020, these 2-day sessions included the opportunity for hands-on practice using CareNav, reviewing new work processes, creating Caregiver Action Plans and service authorizations, generating reports, and using the library of resources available within CareNav. The implementation team provided extensive individual coaching and problem solving to the sites as the implementation proceeded.

#### Executing

**Figure 7:**
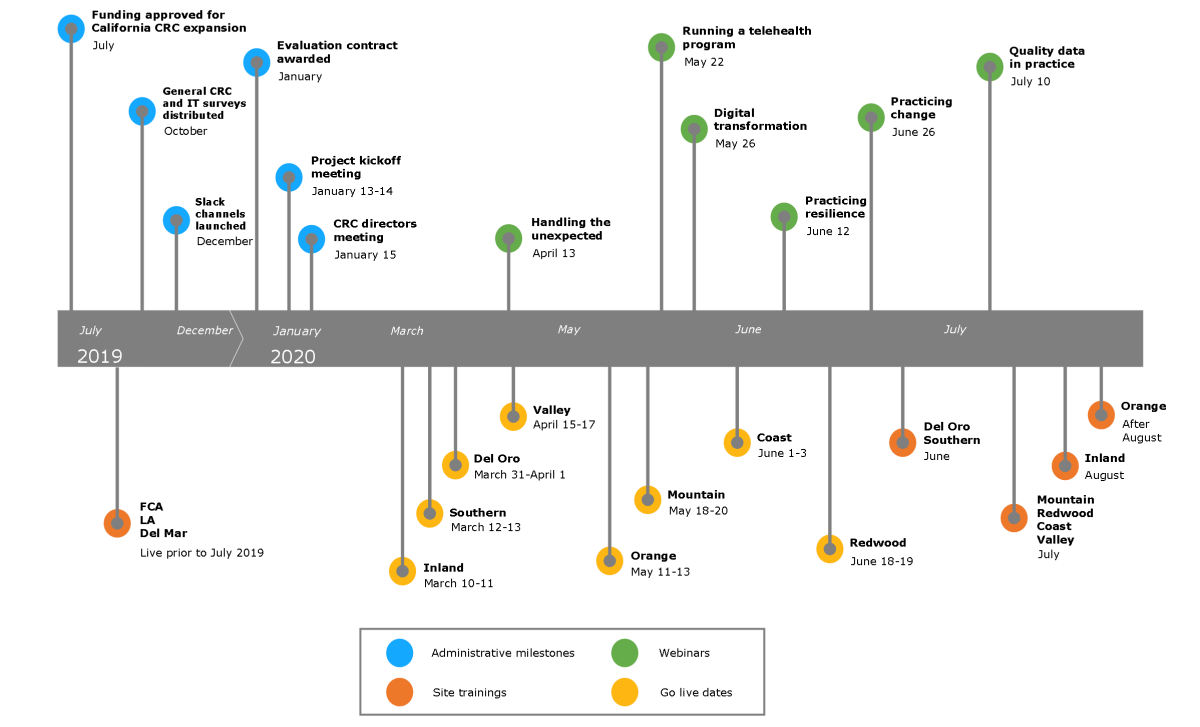
Project timeline. CRC: Caregiver Resource Center; FCA: Family Caregiver Alliance; LA: Los Angeles.

Implementation, including all activities related to training, communication, and installation of CareNav at the 8 sites began in March 2020, and the last site was onboarded in late July 2020 (see timeline in Figure 7). The initial implementation schedule was affected by delays in finalizing state contracts, fund transfers, host agency site requirements for hiring staff, and information technology issues to enable the process to commence. In March 2020, owing to shelter-in-place and physical distancing measures to slow the spread of the COVID-19 pandemic, the sites responded by moving to remote operations with staff working from home.

##### Training and Communication

In addition to the statewide launch meeting followed by training at each individual site, regular statewide web-based training was organized to address best practices, quality improvements, and other topics of broad interest. Owing to state contracting delays, the overall implementation schedule lagged. In some cases, training occurred in March 2020, with delays occurring in technology deployment until July 2020. This necessitated refresher training and ongoing support. Overall, the training was viewed by interview participants as excellent, responsive, customized, and personal.

##### Technology Deployment and Support

The technology team developed and implemented a sophisticated process of CareNav deployment across sites. On the basis of the initial assessment of site-specific technology issues and requirements, they collaborated with staff members to map the data fields from existing data sources to CareNav. System-level requirements for security and interoperability with the existing information technology platform for sites hosted within health systems presented complex challenges with several levels of review and approval. For some sites, particularly those that are more rural, broadband connectivity was an issue for staff working remotely. Following the mapping process, the technology team worked with the sites to perform the data migration and verification. Throughout the process, the implementation team provided both clinical and technical support to assist the sites in making the transition effectively and assisted local staff to become resources for peers.

All sites praised the support received from the implementation team, including their responsiveness and ability to address challenges unique to the site and to their proactive approach to problem solving. It was clear that across all the organizations, both the site directors and staff were highly committed and dedicated to making the implementation a success. Ongoing communication enabled continuous access to the evolving information and knowledge.

The long-term continuous maintenance of the system has been emphasized as a key structural component required to maintain the operational activity of CRCs:

I think it would be great to have a source to go to when things break down...And we’re not gonna go in and fix the system ourselves...So someone maintaining the system is gonna be real important for us to keep it going...that’s part of the whole licensing process- we’re licensed to use the software and support.

Beyond staff training, several interview participants highlighted the importance of training clients on how to use the CareNav system. Currently, the staff members send information to clients about how to log on to the system but thought that this could be enhanced to assist clients who are less experienced with the web environment or face linguistic barriers to a program provided in English. A few sites have identified the importance of marketing and outreach to assist caregivers in finding CRC services to facilitate access:

To me it’s like okay so how do we sell this now and I think it’s a lot it’s gonna be getting them used to it. When my family care navigators schedule video zoom for family consultation...it’s gonna be a lot of that education piece and getting them used to that. I think a change that we have to make internally to help the caregivers use it.

#### Reflecting or Evaluating

##### Overview

Weekly data quality meetings were held with attendance by the evaluation researchers, CareNav application developer, and the FCA Client Services Director. These meetings focused on refining definitions and operationalizing evaluation metrics, defining data filters for the evaluation, identifying and addressing data entry discrepancies overall and by CRC site, and reconciling counts of activities in the evaluation data set with those in reports generated from CareNav. Each quarter, the data quality team met with each of the 11 CRC sites individually to share activity counts and service grant data for comments and any identified data entry issues particular to the site. The team met with the CRC directors approximately 4 times each year to share progress.

Another element of reflection and evaluation is the effectiveness of the training and outcomes for clients and staff. The posttraining survey and in-depth discussions with staff at all CRCs provided insight into the effectiveness of the training, participants’ satisfaction with the process of implementation, as well as early outcomes of CareNav implementation both at the system and the client levels.

Comparing pretraining and posttraining readiness for implementation survey data shows significant increase in staff beliefs about their knowledge how to use CareNav, where to obtain help if needed and their preparedness. The proportion of staff who reported that they understood how to complete an intake and assessment in CareNav significantly increased from 27% before training to 97% after training (*P*<.001 based on the McNemar test). Both before and after training, the staff believed that the system would improve caregiver access to services. Given the high proportion endorsing this survey item in the pretraining and posttraining periods, this change was not statistically significant. Paired 2-tailed *t* test analysis showed significant improvement in feeling prepared to implement the CareNav score (pretraining mean 3.23, SD 1.24 vs posttraining mean 3.63, SD 0.91; *P*=.04) and in knowing where to obtain help (pretraining mean 3.52, SD 1.16 vs posttraining mean 4.32, SD 0.96; *P*<.001). A nearly significant decrease in the mean confidence score was found (pretraining mean 4.39, SD 0.99 vs posttraining mean 4.11, SD 0.93; *P*=.05). It is likely that the training fostered a greater awareness of the system and recognition that it would take time to learn new ways of working and using technology.

Almost all (n=57, 93%) staff reported willingness to adapt current services or provide services using CareNav. All participants will ask for support if they have questions about how to use CareNav, and 55 participants (90%) will offer support to coworkers if they have questions; 52 (85%) participants will ensure new coworkers are educated on how to use CareNav, and 50 participants (82%) will encourage coworkers to use CareNav.

Respondents found the training useful and helpful: 82% (n=50) rated the training as useful, 14% (n=9) were neutral, and 5% (n=3) did not find the training useful. In response to a question about whether the training met their needs, 76% (n=46) of the respondents agreed, 11% (n=7) were neutral, and 13% (n=8) did not feel that the training met their needs. Finally, almost 80% (n=48) felt that they had sufficient time to practice new skills.

##### Satisfaction

Focused interviews explored satisfaction with the system and implementation process. Overall, the interview participants expressed a high level of satisfaction with the implementation process, affirming the vision as important for the next phase of the work of the CRCs and embracing change as a positive force. Interview participants remarked on how easy it was to learn and use the new system and how readily they could see the benefits to their workflow and clients. Many expressed surprise at how well the process had gone despite the unforeseen challenges:

I mean I think for rolling out a new platform of this magnitude, it’s gone amazingly well.

It has been a pretty positive experience. So, I think it’s been easy for me to navigate and I think people who are used to doing everything online appreciate that from the system.

Interview participants from the 2 sites that were the last to implement the program were less satisfied and more skeptical about the long-term benefits. Unlike other sites, these sites encountered system barriers including integration with the information system at the broader host organization and difficulty harmonizing their existing records with the new platform. Despite these frustrations and delays, the sites remained open-minded and were willing to move forward:

I think it just further kind of reaffirms that our staff is flexible and we’ll just roll with it most of the time and yeah it’s frustrating and that sort of thing that we’re going to make the best of it, regardless of the situation and that’s just kind of our attitude about most things in general.

##### Effectiveness of Training

The interview participants recognized that many of the features of the platform were even more relevant in the context of the pandemic and that physical distancing guidelines accelerated adoption by agencies and clients at an unexpected rate. Importantly, throughout the pandemic, CRCs continued to innovate and serve their clients who needed them more than ever:

I think what we’ve all learned that we’ve been very surprised about is we have discovered, so many new opportunities because of the pandemic. And so, our repertoire of services or our availability is expanded. My staff continue to express great satisfaction with their relationships with their clients and, and they are now having satisfying phone conversations where they're having satisfying online support groups, we’re continuing to have our educational workshops and online and people are satisfied with that. So yes there are disappointments and frustrations, but at the same time we’ve discovered so many opportunities. And we’ve become empowered, and we are saying to the community, “We’re here. We’re open we didn't go anywhere, call us, we’re here for you” and so that was a nice surprise, and my staff, myself, the entire organization, we just feel more confident now of knowing how to work remotely, knowing how to use zoom, knowing how to use our new databases, we just feel like, we got it together. I always say to my team they’re rock stars they just impress me every day.

The focused interviews also explored early outcomes of the implementation of the web-based platform, both at the system and client levels. Finally, the participants shared the lessons learned during the process.

##### System Outcomes

The most commonly reported benefits were improved access and convenience. This was particularly salient during the pandemic with staff working from home. Interview participants cited the benefits of having a single paperless location for client data, accessible by any staff member from any location. Family consultants also valued the ability to track services more completely:

It’s allowed us to work remotely, it has allowed at the time of COVID to work safely as a group and still support our caregivers. We’ve been able to continue to offer services. We’ve continued to be able to document and work together.

The directors valued the potential of the system to generate data to evaluate services, improve quality, guide program decisions, and provide evidence for advocacy. At the site level, directors anticipated using the data to guide strategic directions and identify service gaps. Statewide, they valued the potential to aggregate data to inform planning and policy. For example, data on caregivers can inform the implementation of the California Master Plan on Aging:

The other big benefit eventually will be that we will have statewide information data that is consistent across all 11 Caregiver Resource Centers. And to me that's huge because it helps with government planning and policymaking as it relates to caregiving.

A positive consequence of this initiative has been bringing the sites together, with a shared vision to serve caregivers across the entire state, learning from one another and sharing resources. Several directors shared a vision to collaborate in new ways, assuring that caregivers across the state had access to culturally and linguistically congruent resources. For example:

Could we survey our sites to see who has family consultants in different languages? In our County we have a pretty sizable Korean population. We currently don’t have a Korean family consultant, but if another site did, could they serve them? And could we serve if they had a Vietnamese client? In other words, how do we leverage our statewide network around language barriers? I think there’s a potential opportunity to use the statewide network differently with this common platform.

##### Client Outcomes

The greatest client benefit reported was having a platform that clients could access at their convenience, around the clock and from any location. In addition, the consultants found it beneficial that the platform enabled them to tailor resources for specific clients based on their assessment, which allowed for immediate delivery from the web-based library. Clients could also access information about authorized services, such as respite or counseling on the web. Several consultants voiced their advantage to clients with disabilities who find web-based navigation more accessible than other alternatives:

I have clients with disabilities, for example, I had a few who were completely deaf and—we communicated through (CareNav) and through email. So, that was a wonderful tool for her because she said in the past it was really challenging to get services.

The new platform provided an avenue for clients to connect during the COVID-19 pandemic and address social isolation in a way that had not been previously available:

One of our staff members said, “my online Spanish support group are really having a hard time with the isolation and they so appreciate being together at least once a month, but they want to meet two times. Can I do my support group two times a month?” I said of course!

##### Lessons Learned

The most commonly stated lesson was *patience*. All the interview participants reported resilience and flexibility as they approached and engaged in the implementation process. Many affirmed the positive nature of the change and a commitment to ongoing learning associated with this platform enhancement. They expressed heightened appreciation for the importance of communication and collaboration within sites, across sites, and in the community. For those on the clinical and technical support teams, they recognized in even greater detail the variability and diversity across the sites:

Having to realize that people really think differently, and they learn at different speeds, and they communicate differently, both individuals and as organizations, and the variability in even their data or how they store their data and how they manage their programs. Yeah, I think there was just a little bit of variability everywhere.

## Discussion

### Principal Findings

Our findings suggest 4 factors as essential underpinnings of successful implementation, with implications for future replication [29]. First, key informants emphasized the importance of effective leadership as a key inner-setting component. The participants cited creativity, perseverance, unwavering vision, clear communication, relevant expertise, and deep knowledge of all aspects of the intervention and the inner and outer settings. Effective leadership facilitates a positive implementation climate, which in turn enhances implementation effectiveness [30]. Beyond the inner setting, leadership was described as continually interacting and potentially affecting other CFIR domains [31]. The CareNav implementation team has long tenure in the system and a history of successful implementation of other prior initiatives across the CRC system that may have increased staff self-efficacy. The expertise of program managers, information technology staff, and site directors and staff, as well as leaders’ deep knowledge of CareNav and the CRCs outside networks contributed to continuous interaction with stakeholders throughout the technology implementation process [31]. The leaders’ deep understanding of unique CRC site-level structures and processes was described as particularly important for enhancing both customized and overall implementation efforts. These findings echo recent technology implementation studies in multisite settings, highlighting the lack of senior leadership endorsement as impeding successful implementation [32].

Second, the interview participants stressed the key role of training and ongoing support to increase their self-efficacy using the technology platform. Ongoing training and support were suggested as effective strategies to attract and involve key stakeholders in implementing or using the innovation [33,34]. Our results in the implementation process domain provide insight into the participants’ thoughts on the ongoing training performed. The implementation leadership team gathered information about the system user experience from the training and applied the learning for system refinement and quality improvement. As reflected in both the surveys and interviews, participants appreciated the pace and cadence of the training as well as the opportunity to provide feedback. This feedback loop, in turn, improved platform usability, further inspiring staff self-efficacy. Of note, this 2-way communication for continuous improvement is ongoing with quarterly meetings of the QP development team, program evaluators, and CareNav end users to review program productivity data and share issues, concerns, and feedback—providing an open forum for problem solving in the future.

Third, CareNav represented culture change in two major ways: by bringing together decentralized sites and by introducing novel technology that provides customization, round-the-clock availability, and enhanced workflow for staff. In addition, CareNav reflects culture changes societally. In both surveys and interviews, participants expressed a high degree of willingness to change and an openness to new approaches to delivering and documenting interactions with caregivers. This willingness to adopt technology was a key factor in the rapid and successful deployment of the platform.

Finally, the interview participants raised a critical issue of ongoing funding to support the CareNav initiative going forward. Over the last few decades, state funding for the CRC system has waxed and waned, along with the political will to support family caregivers. In the leanest years, CRCs functioned largely as independent entities, drawing on local funding and scaling back services accordingly. In the current phase of funding, state-level investment for CareNav was significant, covering the technology roll out across 11 sites and supporting the QP CareNav development team to provide ongoing technical support, system refinement, reporting capability, and building system elements that expand the functionality of the user interface of the portal through which caregivers can complete intake and assessment forms and access information around the clock. Without ongoing funds to support this vital work, the impact of this initial investment will be vastly diminished, and none of the stakeholders (caregivers, CRCs, and funders) will be able to capitalize on its full potential. Indeed, the long-term benefits of a uniform assessment, data collection, and reporting system are yet to fully materialize, with great potential to disseminate interventions to support family caregivers in the future.

### Context of the COVID-19 Pandemic

This study echoes the recent implementation literature underscoring the COVID-19 pandemic as an outer-setting construct inconclusively affecting implementation [35-37]. In fact, the pandemic may have facilitated and accelerated the technology implementation process with significant effects on CareNav characteristics, the inner setting, characteristics of the CRC staff, and the outer setting. CareNav proved to be an agile system during the unexpected national and global circumstances of the COVID-19 pandemic and showed promise of helpfulness in sudden onset or unforeseen conditions, especially in California, given the likelihood of natural disasters such as earthquakes or fires and their impacts on caregivers and the caregiving role. Indeed, the vision for a shared technology platform proved to be prescient as the COVID-19 pandemic gained momentum and the CRCs were able to respond quickly to provide uninterrupted services to their clients by ramping up outreach and adapting supports for caregivers. As examples related to the inner setting, the circumstances of the pandemic may have improved the organizational will to implement by spotlighting the perceived benefits of the technology platform, given its ability to support web-based work; improved the implementation climate through increased staff enthusiasm for the platform; increased staff patience to follow through the stages and steps of the implementation process; reduced hesitation about changing to the technology platform; and increased the need for cross-site communication to best serve the needs of clients. Related to the outer setting, CRC staff members were acutely aware of the toll the pandemic took on their clients, as their needs increased and access to many services and supports—including respite care, adult day care, and institutional placement—became more limited.

At the same time, CRC staff members themselves experienced added strain, juggling their own competing demands outside work, covering for one another owing to illness, coping with staff turnover, and pivoting to remote work from home to provide web-based family consultations to their clients. These factors may have either increased or decreased the willingness of the staff to use the CareNav platform to do their work in new ways, their readiness for change, and their perceived need to expand services. Moreover, the pandemic might have slowed the ability to integrate CareNav with other systems, specifically for sites hosted within a larger health care system who were themselves focusing on care delivery priorities. Similarly, the implementation of other web-based technologies encountered significant barriers, explained by time constraints and competing priorities associated with the COVID-19 pandemic [38]. Effective leadership engagement, as demonstrated in our study, might have played a mediating role in buffering the COVID-19 pandemic slowing effect by maintaining commitment and resources to the implementation and ultimately reducing behavioral resistance to change [35,36,39]. Taken together, these factors must be considered in the interpretation of the findings of this evaluation, particularly in light of the perceived success of the implementation from the staff perspective.

### Comparison With Prior Work

This implementation evaluation also offers the knowledge needed to address gaps in prior research. A systematic review of internet-based supportive interventions for caregivers of patients with dementia recommended individual tailoring of supports for the success of digital interventions for caregivers [23]. Underlying this recommendation is the heterogeneity of web-based support systems regarding intervention type, dosage (amount of time spent on the web), and duration, which affect intervention effectiveness, feasibility, and quality [23,40]. In addition, caregivers use web-based supports targeting caregiving for distinct illnesses such as cancer [41], dementia [10], posttraumatic stress disorder [20], and psychosis [21,22] suggesting illness-specific web-based support needs for caregivers. As described, CareNav enables either self-administered or staff-administered caregiver and care recipient assessment, appraisal of current resources, delineation of priority supports, and development of a care plan to contract appropriate services such as respite, educational sessions, support groups, and vouchers for legal aid or counseling across all health conditions, providing a more comprehensive resource for caregivers. These services can be individually tailored to caregivers and illness-specific caregiving needs. CareNav includes a tailored library of resources for caregivers based on the assessment, with the ability to link to resources such as the World Health Organization’s iSupport site [10].

Studies in the past have shown that web-based support can increase confidence, self-efficacy, and self-esteem and reduce depression and strain among caregivers [14,23,42]. This study showed that the capacity to enhance access, convenience, and customized support for clients held a universal appeal for CRC staff. CRC sites highly valued the potential of the data collected in CareNav to provide real-time feedback on who they were serving and the effectiveness of their interventions. Future analyses should explore changes in client confidence and mental health outcomes. As users gain expertise and competence, they will likely see greater benefits to full use of the CareNav platform features, enhancing client engagement and improving decision-making for care consultants, particularly for clients at the highest risk for negative outcomes.

### Limitations

This study was an evaluation of an implementation process in established CRCs across the state. There were trade-offs in data collection and reporting to protect the anonymity of participants who could otherwise be readily identified. Therefore, this study does not provide demographic characteristics of participants nor is it able to link findings to the specific roles of staff. However, the decision to permit anonymity facilitated broader participation and a more complete perspective on implementation, as evidenced by the high participation rate of staff in both the surveys and interviews. We applied the CFIR during the data analysis stages of the implementation and did not use the framework to its full potential in guiding implementation design. This study focused on the implementation process and its immediate impact on staff and clients, and did not examine changes in caregiver health and well-being.

### Conclusions

In this study, we applied the CFIR [29] to examine the implementation of a web-based platform to engage and support family caregivers across 11 regional sites in California, the most populous state in the United States. The findings identified factors that contributed to success and were relevant for future scaling and dissemination of this innovative platform designed to support global assessment, intervention, and service delivery to address the unmet needs of family caregivers. Importantly, the platform is accessible to both staff and clients regardless of geographic region or care recipient medical condition, and supports timely and local responses to caregiver priorities. Using the CFIR facilitated a more systematic understanding of the multilevel experience of technology implementation across multiple sites. Future implementation projects would benefit from using the CFIR to organize implementation strategies and identify potential blind spots in planning.

As noted, the value of long-term care provided by family caregivers eclipses that provided by government funding [2]. Caregivers have become indispensable in health care delivery. Although their contributions have remained largely invisible, CareNav elevates caregivers’ contributions as part of the fabric of health care. The global assessment of the caregiver and the ability to respond to the priority of the caregiver in real time with information or referral responds to myriad health-related urgencies and imbeds caregivers as elemental in the fluid and ongoing process of care. CareNav additionally supports caregivers in the community. Studies show that the caregiver community is helpful over and above the benefits related to caregivers’ coping [43]. Information gathering, reminiscing, legacy building, and giving back to the community of caregivers maintain the spirit of commitment [42,43]. Although in our study, the mechanism of culture change is primarily associated with modernizing business practices using technology, the CRC vision and long-range outcomes align with broader societal implications of acknowledging and supporting caregivers as a crucial link in providing supports across communities.

Among the 5 bold goals of California’s Master Plan on Aging for 2030 is *caregiving that works* [44]. The California CRCs took on the impressive goal to harmonize their information technology platform and implement the new platform across the state in all 11 sites in 6 months. Other state plans, such as those of New York, have similar priorities for caregivers and CRCs [45]. Future studies could compare California state data with both other states and national populations. This could produce greater knowledge of the similarities and differences in the support needs for the nation’s caregivers. With the opportunity for longitudinal analysis, future studies could examine the predictive factors of intake for negative caregiver outcomes, facilitating earlier interventions. Further analysis of use and outcomes could elucidate which services are most helpful for subpopulations of caregivers.

The next phase of implementation should involve developing a deeper understanding of the rich information available through CareNav, determining both site-specific and statewide reports that would be most helpful in evaluating adoption and dissemination of the programs into the community, effectiveness and gaps in service, quality of delivery, and the impact of the services and supports on caregiver outcomes. The data hold the power to drive individual and system changes. At the individual level, the assessment can be the basis for determining risk and matching services to caregiver needs. At the system level, the data can drive strategy for priority program development, funding, and advocacy. Future studies should evaluate the impact of the CRC system on caregiver health and well-being, as well as develop a deeper characterization of the trajectory of caregiving and how interventions can improve outcomes for caregivers and the persons in their care.

A determined and committed group of leaders and staff dedicated to improving the lives of caregivers began a journey together. They are well on their way to actualizing a vision for the future of caregivers in California.

## Abbreviations

CFIR: Consolidated Framework for Implementation Research
CRC: Caregiver Resource Center
FCA: Family Caregiver Alliance
QP: Quality Process
